# Transcutaneous Spinal Stimulation Combined with Locomotor Training Improves Functional Outcomes in a Child with Cerebral Palsy: A Case Study

**DOI:** 10.3390/children11121439

**Published:** 2024-11-26

**Authors:** Darryn Atkinson, Kristen Barta, Fabian Bizama, Hazel Anderson, Sheila Brose, Dimitry G Sayenko

**Affiliations:** 1 School of Rehabilitation Sciences, Doctor of Physical Therapy Program, South College, 616 Marriott Drive, Nashville, TN 37214, USA; 2 School of Physical Therapy, University of North Texas, 3500 Camp Bowie Blvd, Fort Worth, TX 76107, USA; 3 Doctor of Physical Therapy Program, University of St. Augustine for Health Sciences, 5401 La Crosse Ave, Austin, TX 78739, USA; 4 Department of Neurosurgery, Houston Methodist Hospital/Research, 6565 Fannin St, Houston, TX 77030, USA

**Keywords:** cerebral palsy, neuromodulation, activities-based locomotor training, transcutaneous spinal stimulation

## Abstract

Background and Purpose: activities-based locomotor training (AB-LT) is a restorative therapeutic approach to the treatment of movement deficits in people with non-progressive neurological conditions, including cerebral palsy (CP). Transcutaneous spinal stimulation (TSS) is an emerging tool in the rehabilitation of individuals with sensorimotor deficits caused by neurological dysfunction. This non-invasive technique delivers electrical stimulation over the spinal cord, leading to the modulation of spinal sensorimotor networks. TSS has been used in combination with AB-LT and has been shown to improve muscle activation patterns and enhance motor recovery. However, there are no published studies comparing AB-LT + TSS to AB-LT alone in children with CP. The purpose of this case study was to compare the impact of AB-LT alone versus AB-LT combined with TSS on functional movement and quality of life in a child with CP. Methods: A 13-year-old male with quadriplegic CP participated in this pilot study. He was classified in the Gross Motor Function Classification System (GMFCS) at Level III. He completed 20 sessions of AB-LT (5x/week), then a 2-week washout period, followed by 20 sessions of body-AB-LT + TSS. Treatment sessions consisted of 1 h of locomotor training with body weight support and manual facilitation and 30 min of overground play-based activities. TSS was applied using the RTI Xcite^®^, with stimulation at the T11 and L1 vertebral levels. Assessments including the Gross Motor Function Measure (GMFM), 10-m walk test (10 MWT), and Pediatric Balance Scale (PBS) were performed, while spatiotemporal gait parameters were assessed using the Zeno Walkway^®^. All assessments were performed at three time points: before and after AB-LT, as well as after AB-LT + TSS. OUTCOMES: After 19/20 sessions of AB-LT alone, the participant showed modest improvements in the GMFM scores (from 86.32 to 88), 10 MWT speed (from 1.05 m/s to 1.1 m/s), and PBS scores (from 40 to 42). Following the AB-LT combined with TSS, scores improved to an even greater extent compared with AB-LT alone, with the GMFM increasing to 93.7, 10 MWT speed to 1.43 m/s, and PBS to 44. The most significant gains were observed in the GMFM and 10 MWT. Additionally, improvements were noted across all spatiotemporal gait parameters, particularly at faster walking speeds. Perhaps most notably, the child transitioned from the GMFCS level III to level II by the end of the study. Discussion: Higher frequency and intensity interventions aimed at promoting neuroplasticity to improve movement quality in children with CP are emerging as a promising alternative to traditional physical therapy approaches. This case study highlights the potential of TSS to augment neuroplasticity-driven treatment approaches, leading to improvements in neuromotor function in children with CP. These findings suggest that TSS could be a valuable addition to rehabilitation strategies, warranting further research to explore its efficacy in larger populations.

## 1. Introduction

Cerebral Palsy (CP) is a nonprogressive neurological condition caused by damage or abnormal development of the brain. While motor and sensory impairments persist throughout the lifespan, their manifestations may change over time [1]. Individuals with CP often experience associated conditions including cognitive impairments, communication difficulties, seizures, visual or hearing deficits, and musculoskeletal complications such as spinal deformities and osteoarthritis [2]. According to the Centers for Disease Control, the global prevalence of CP ranges from one to four per 1000 children, with higher rates observed in infants with low birth weight [3].

There a five main types of CP: spastic, dyskinetic, ataxic, hypotonic, and mixed. Spastic CP accounts for approximately 80% of all diagnosed cases and is characterized by hypertonicity that may affect the lower extremities, one side of the body, or the entire body, including the trunk. Spasticity can lead to contractures, stiffness, and difficulty with active movement. Dyskinetic CP, also known as athetoid, presents with fluctuating muscle tone causing involuntary movements during active and passive motion. Ataxic CP is associated with dysfunction in coordination, balance, and voluntary movements. Hypotonic CP represents the opposite spectrum of spastic CP and is characterized by lower muscle tone throughout the body, resulting in decreased muscle activation and overall instability. Finally, mixed CP occurs when the damage is not isolated to one area and multiple clinical presentations are seen within the same individual. All types of CP may be accompanied by pain, gastrointestinal issues, and respiratory complications, ultimately affecting functional activities, participation, and quality of life [2,4]

Children with CP may experience a variety of neurological impairments, with motor dysfunction being the most common. This dysfunction can range from hypotonicity to hypertonicity in the trunk and extremities [4]. These motor impairments often lead to musculoskeletal issues and functional limitations, affecting activities such as crawling and walking. Developing a comprehensive rehabilitation program is essential to promote movement and reduce complications, contributing to the overall health and well-being of children with CP [5].

Activities-based locomotor training (AB-LT) is a restorative therapeutic approach that aims to improve neuromuscular capacity through the application of the principles of neuroplasticity, to promote improved functional mobility in both children and adults with non-progressive neurological conditions, including CP. Neuroplasticity, the central nervous system’s ability to reorganize itself in response to experiences, plays a crucial role in this approach. Depending on environmental factors, neuroplastic changes can be adaptive or maladaptive [6]. AB-LT aims to improve neuromuscular capacity by harnessing the principles of neuroplasticity. Key elements of AB-LT encompass intensity, repetition, and salience, all of which are essential for driving adaptive neuroplasticity, which has previously been demonstrated in children with CP [7,8].

Transcutaneous spinal stimulation (TSS) is an emerging rehabilitation tool for individuals with sensorimotor deficits caused by neurological dysfunction. TSS non-invasively delivers electrical pulses to the dorsal spinal cord activating spinal circuitry projecting to targeted muscles [9]. This stimulation can produce motor responses via excitation of Ia afferents synapsing on α-motoneurons and can also activate other neural structures within the spinal cord, including interneurons, ascending sensory fibers, and descending motor tracts [10,11,12,13,14]. TSS has been used in conjunction with AB-LT in adults and children with neurological diagnoses and shown to improve muscle activation patterns as well as augment the recovery of motor function [15]. Several research groups have demonstrated the feasibility of using TSS to modulate the excitability of spinal sensorimotor networks in individuals with spinal cord injury (SCI), multiple sclerosis, and CP, facilitating both voluntary and autonomic functions [10,16,17,18,19,20,21]. These findings provide strong evidence that the activation of sub-functional longitudinal fibers within the spinal cord, along with the emerging responsiveness of spinal networks to descending commands and sensory inputs, are the primary mechanisms through which TSS restores sensorimotor function following severe neurological conditions [13].

Considering the mechanistic effects of spinal stimulation in general, and the non-invasive nature of TSS in particular, it is plausible that TSS could improve sensorimotor function in children with movement deficits due to CP [19]. Therefore, the overall objective of this feasibility and safety study was to investigate the effect of TSS, as a non-invasive neuromodulation approach, on neuromuscular capacity in a child with CP. We hypothesized that TSS-induced modulation of spinal neural circuitry would improve functional mobility and ambulation.

## 2. Methods

### 2.1. Case Description

A thirteen-year-old male participant with spastic quadriplegic CP, Gross Motor Function Classification System—Expanded and Revised (GMFCS- E&R) level III, participated in this study. The participant was born prematurely at 27 weeks. At age six, he was diagnosed with CP and received therapeutic assistance at an institution for children with neurological deficits. At nine years of age, he was adopted and brought to the United States, where he was diagnosed with CP, left clubfoot, and bilateral hip dysplasia. From age nine to thirteen, he received PT services intermittently. At age ten, he was recruited to participate in AB-LT five days per week. He participated for approximately 4 months until the program was closed due to the COVID-19 pandemic. The participant did not receive PT services over the next two years, until age 13, when he returned to AB-LT 6 months post left clubfoot correction surgery. He required assistance with transfers and standing balance, used a wheelchair for long distances, and a left ankle foot orthosis (AFO) and posterior rolling walker for ambulation in the home. He resumed participation in AB-LT, completing 61 sessions and two 4-week breaks, prior to enrollment in the current study. He progressed to using forearm crutches in the community while bringing his wheelchair as a backup for longer distances. He was able to take steps without his crutches at home, using furniture for support as needed, along with ascending and descending stairs using one handrail at home and in the community.

The participant’s mother provided written informed consent through the University of St. Augustine for Health Sciences, IRB # PT-1001-307.

### 2.2. Study Design

The participant completed 19/20 sessions of AB-LT (5×/week), followed by a 2-week washout period, and then 20 sessions of body-AB-LT + TSS. Treatment sessions were 1.5 h long and consisted of (a) 1 h of locomotor training (LT) in which he walked on a treadmill with body weight support and manual facilitation at the trunk and lower extremities as needed and (b) 30 min of overground play-based activities. Assessments were performed prior to AB-LT, after AB-LT, and again after AB-LT + TSS. Assessments included the following: Pediatric Balance Scale (PBS), 10 Meter Walk Test (10 MWT), the Zeno Walkway^®^ (ProtoKinetics, Havertown, PA, USA), and the Gross Motor Function Measure (GMFM).

### 2.3. Activities-Based Locomotor Training

Activities-based locomotor training was delivered following the principles of locomotor training [22]. The body weight-supported treadmill component of each session consisted of 3 different modes of training: step retraining (SR), step adaptability (SA), and stand adaptability (STA). During SR, the participant steps at a natural walking speed with body weight support, manual facilitation, and verbal cues applied to optimize kinematics during stepping. The goal during SR is to present the nervous system with optimal sensory input related to stepping to facilitate postural control and coordinated stepping. Step adaptability focuses on independent postural control and stepping. During SA, body weight support and treadmill speed may be decreased to allow the participant to step without manual assistance. During STA, the focus is again independence; the goal in this phase of training is upright posture in standing, without body weight support and manual assistance. Following the body weight-supported treadmill component, the participant was engaged in overground activities, with the same goal of uncompensated balance/mobility. This typically occurs in the form of a game or activity in order to create motivation for the child.

### 2.4. Transcutaneous Spinal Stimulation

**Figure 1:**
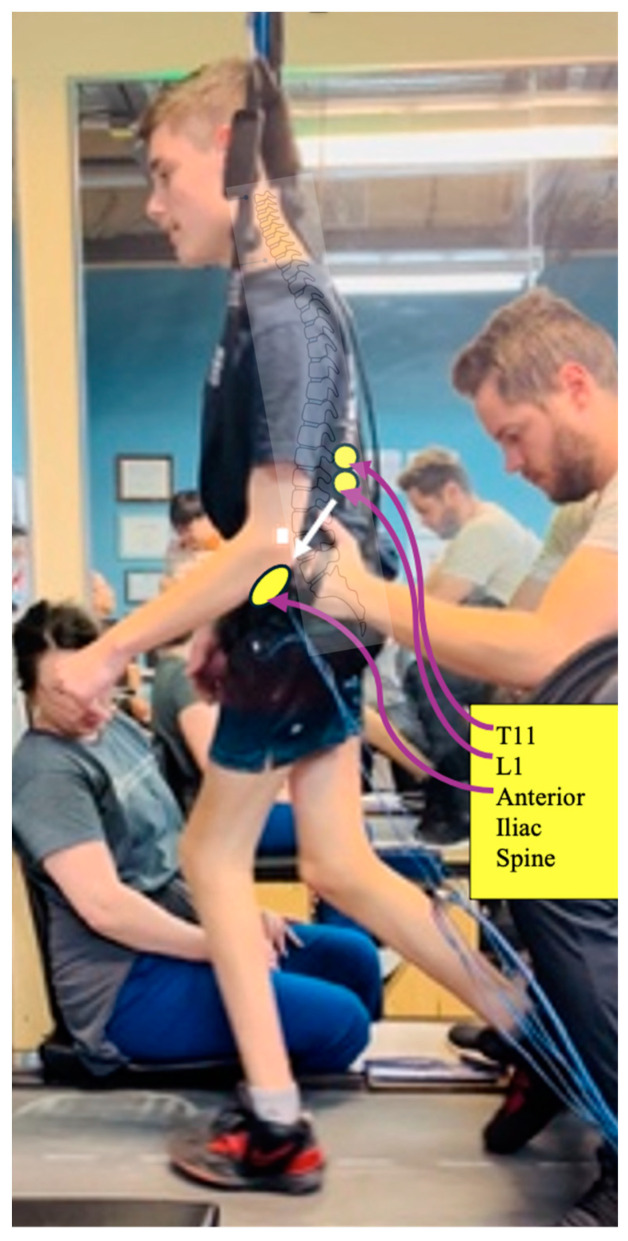
P1 in the body-weight support treadmill environment with TSS. Electrode placement (yellow): two pairs of electrodes were used, with one of each pair (one-inch round electrodes) placed over the T11 and L1 spinous processes and the other (2- × 3-inch oval electrodes) over each anterior superior iliac crest. Then, the pelvic and thoracic harnesses were applied.

Transcutaneous spinal stimulation was applied using the RTI Xcite^®^ stimulator (Restorative Therapies, Inc., Nottingham, MD, USA) at the T11-T12 and L1-L2 intravertebral spaces. Two pairs of electrodes were used (one-inch round electrodes placed over the T11-T12 and L1-L2 intervertebral spaces and (2- × 3-inch oval electrodes) for each anterior superior iliac crest (Figure 1). The stimulation was delivered as biphasic pulses, with a pulse width of 500 µs pulses at a frequency of 20 Hz. During the TM portion of each session, stimulation was applied continuously, with the intensity adjusted daily to the maximum level tolerated by the participant.

### 2.5. Assessments

#### 2.5.1. Pediatric Balance Scale

The Pediatric Balance Scale is a balance measure using a Likert scale from 0–4 to assess static and dynamic stability. The measure ranges from a score of zero to 56 with a higher score indicating better postural stability. The measure has excellent concurrent and predictive validity with the Gross Motor Function Measurement at baseline (r = 0.92–0.95) and (r = 0.90–0.92), respectively, in the CP population. The Minimal Detectable Change is 1.59 points, and the Minimally Clinical Importance Difference is 5.83 points [23]. The assessment was completed in its entirety for the participant at the scheduled testing times.

#### 2.5.2. Ten Meter Walk Test

The 10 MWT is used to assess gait velocity overground and was set up as a marked runway of 10 m on a smooth, flat surface. The middle six meters were timed using a stopwatch with the initial and final two meters serving as an area for acceleration and deceleration as this setup has shown to be optimal in the neurologic population [24]. The measure shows excellent test–retest reliability (ICC = 0.91) in children with neurological conditions [25].

#### 2.5.3. Zeno Walkway^®^

The Zeno Walkway System was used to measure the participant’s velocity and gait pattern. Computerized walkway mats are valid and reliable tools for measuring the spatiotemporal parameters of gait [26]. Electronic walkway systems have been shown to have high test–retest reliability when used in a short-term time span for children with CP [27]. To measure gait velocity using the Zeno Walkway System, the participant was instructed to walk across the mat at his preferred walking speed. He was instructed to start at one end and walk across and off the mat to the other end, turn, and stand until instructed to walk the length of the mat again. This setup allowed for gait acceleration and deceleration distances to occur outside of the walkway. He completed three trials, and the average was used for all spatiotemporal parameters.

#### 2.5.4. GMFM—88

The Gross Motor Function Measure—88 (GMFM) is a reliable criterion-referenced outcome measure, highly responsive to functional gross motor changes over time or to compare changes due to intervention in children with CP [28]. Comprising 88 items, there are five dimensions related to gross motor skills: (A) Lying and Rolling, (B) Sitting, (C) Crawling and Kneeling, (D) Standing, and (E) Walking, Jumping, and Running [28]. Each item is scored using a 4-point ordinal scale with (0) = does not initiate, (1) = initiates, (2) = partially completes, and (3) = completes. Items not tested (NT) are labeled NT. The total points for each dimension are calculated and assessed using a scale of 0–100, with higher percentage scores reflecting higher gross motor function. The average of the five dimensions denoted the Total GMFM percentage score.

## 3. Results

### 3.1. Clinical Presentation

Upon initial observation, the participant walked into the clinic with forearm crutches. He presented with impairments that significantly affected his functional skills, mobility, and participation at home and in his community, including increased low extremities (LE) tone in hip adductors, hamstrings, and gastrocnemius, with the left side more affected than the right. He had limited range of motion in his hips, knees, and ankles and bilateral lower extremity more than upper extremity weakness, with the left side again more affected than the right. Other impairments included decreased standing balance, postural stability, bilateral LE coordination, and proprioception/somatosensory awareness. He also had moderate to severe orthopedic deformities, including bilateral femoral anteversion, left more than right, genu valgus, and left foot equinovarus with forefoot adduction. These structural deformities affected his mobility and gait at home and in the community, increasing his fall risk. He had an AFO for left LE support, which he did not wear consistently. He could ambulate distances of 300 feet with forearm crutches in a four-point gait pattern, demonstrating LE scissoring and landing on the lateral aspect of the left foot. At home, he reported that he ambulated using furniture as needed for balance. He used forearm crutches for short distances, a posterior walker for medium distances, and a manual wheelchair for longer distances, or when navigating different terrains in the community. At the time of the initial physical therapy evaluation, he was not taking any medication nor had he received Botox injections for spasticity management.

### 3.2. Initial Assessment

**Figure 2:**
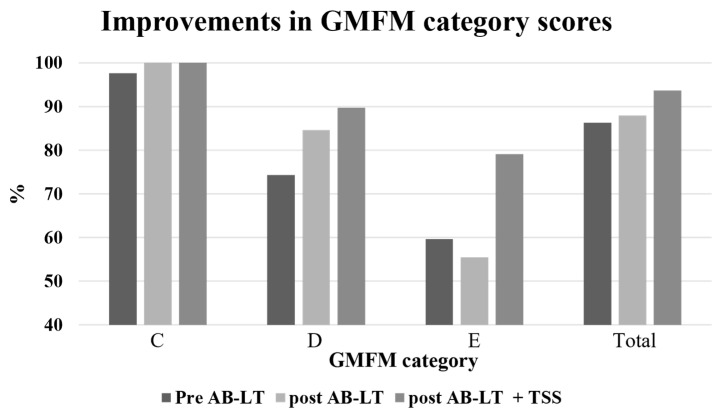
Improvements in GMFM category scores. GMFM scores for categories C, D, E, and total score for each time point: Pre-AB-LT = prior to activities-based locomotor training, post-AB-LT = following AB-LT training, post-AB-LT + TSS = following AB-LT with transcutaneous spinal stimulation.

**Figure 3:**
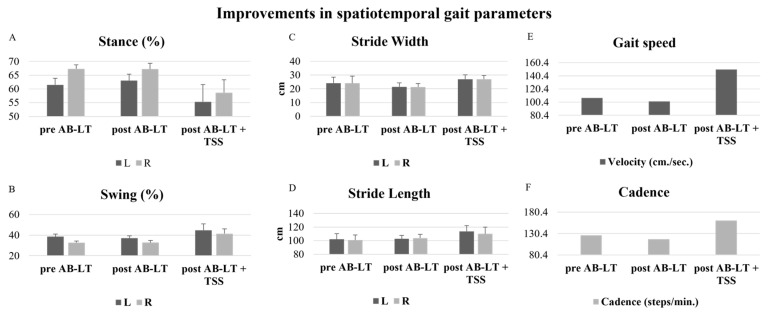
Improvements in spatiotemporal gait parameters. Panels A and F give values for (**A**) percentage of time in stance, (**B**) percentage of time in swing, (**C**) stride width, (**D**) stride length, (**E**) Gait speed, and (**F**) cadence, before AB-LT (pre AB-LT), after AB_LT (post AB-LT), and after AB-LT with TSS (post AB-LT + TSS). L and R = left and right, respectively.

On initial assessment, the participant scored 40/56 on the PBS and demonstrated walking speeds of 0.71 m/s and 1.05 m/s on the 10 MWT for self-selected and fast walking, respectively (Table 1). The participant scored the following on the five dimensions of the GMFM: A = 100%, B = 100%, C = 97.6%, D = 74.3%, and E = 59.7%, with an overall score of 86.32% (Table 1, Figure 2). Spatiotemporal measures of gait were obtained using the Zeno Walkway^®^ (ProtoKinetics, Havertown, PA, USA) and included stride length, stride width, stance % (left and right), swing % (left and right), velocity, and cadence (Table 1, Figure 3).

### 3.3. Activities-Based Locomotor Training Without TSS

The participant completed 19/20 AB-LT sessions, with an average total treadmill time of 52.97 min (Table 2). During this time, the average body-weight support was 35.53% of total body weight. The average time spent in SR was 15.22 min, with the participant stepping at speeds from 1.2–1.8 miles per hour (mph). The average time spent in SA was 11.64 min, at speeds from 1.2–1.5 mph. The average total time spent stepping (SR + SA) was 26.86. The average time spent in STA was 25.22 min. Overground interventions incorporated functionally relevant and challenging tasks into play, specifically focusing on improving upright posture, static and dynamic standing balance, and gait while minimizing compensations such as upper extremity weight bearing. The patient was provided verbal and visual cues, minimal assistance for balance, and facilitation for knee extension. Most activities were performed in front of a mirror for visual feedback.

### 3.4. Post AB-LT Assessment

Following 19/20 sessions of AB-LT, the participant demonstrated improvements in all outcome measures. His PBS score improved from 40 to 42/56, and his 10 MWT times improved from 0.71 to 0.75 m/s, and from 1.05 to 1.1 m/s for self-selected and fast speeds, respectively (Table 1). The GMFM scores for each domain following AB-LT were A = 100%, B = 100%, C = 100%, D = 84.6%, and E = 55.5%, with a total score of 88% (Table 1, Figure 2). Notable changes in spatiotemporal gait values following AB-LT included an increase in stride length coupled with a decrease in stride length variability, with stride length increasing from 76.36 ± 18.40 cm before AB-LT to 84.69 ± 7.92 cm after AB-LT. After AB-LT, his cadence decreased to 103.03 steps/minute, compared to 113.98 steps/minute at the initial assessment (Table 1, Figure 3).

### 3.5. Activities-Based Locomotor Training with TSS

The participant completed 20/20 AB-LT+TSS sessions, with an average total treadmill time of 56.70 min (Table 2). During this time, the average body-weight support was 28.25% of total body weight. The average time spent in SR was 20.13 min, with the participant stepping at speeds from 1.6–2.0 miles per hour (mph). The average time spent in SA was 12.75 min, with the participant stepping at speeds from 1.4–1.8 mph. The average total time spent stepping was 26.86, and the average time spent in STA was 32.88 min. TSS was delivered continuously while on the treadmill. The average stimulation intensity at T11 was 24.9 ± 8.74 mA, with a range between 13 and 45 mA. The average stimulation intensity at L1 was 21.5 ± 6.01 mA, with a range between 15 and 35 mA. Overground interventions during AB-LT + TSS continued to focus on posture, gait, and dynamic balance and progressed as the participant’s ability to perform improved.

### 3.6. Post AB-LT + TSS Assessment

Following 20 sessions of AB-LT + TSS, the participant demonstrated improvements in all outcome measures as compared to following AB-LT alone. His PBS score improved from 42 to 44/56, and his 10 MWT times improved from 0.75 to 0.78 m/s, and from 1.1 to 1.43 m/s for self-selected and fast speeds, respectively (Table 1). Gross Motor Function scores for each domain following AB-LT were A = 100%, B = 100%, C = 100%, D = 89.7%, and E = 79.1%, with a total score of 93.7% (Table 1, Figure 2). Notable changes in spatiotemporal gait values during self-selected walking speed following AB-LT + TSS included an increase in stride width and a decrease in stride width variability, from 6.94 ± 21.21 cm after AB-LT, to 21.50 ± 4.20 cm after AB-LT + TSS (Table 1, Figure 3). During fast walking speeds, the percentage of the step cycle in the stance phase decreased following AB-LT + TSS, with a corresponding increase in the percentage of the cycle spent in the swing phase. After AB-LT + TSS, time in stance decreased from 63.04 ± 2.32% on the left and 67.20 ± 2.09% on the right to 55.33 ± 6.24% on the left and 58.64 ± 4.71% on the right. During fast walking, stride length increased as compared with post-AB-LT assessment, increasing from 102.71 ± 5.04 cm after AB-LT to 113.66 ± 8.61 cm after AB-LT + TSS. At fast walking speeds, both velocity and cadence increased following AB-LT + TSS, with velocity increasing from 101.43 cm/sec after AB-LT to 150.13 cm/sec after AB-LT + TSS and cadence increasing from 117.16 steps/minute after AB-LT to 160.90 steps/minute after AB-LT + TSS (Table 1, Figure 3).

## 4. Discussion

The purpose of this case study was to compare the impact of AB-LT alone versus AB-LT+ TSS on functional movement ability in a child with CP. While intensive interventions such as AB-LT have been shown to improve posture and functional mobility in children with CP [29,30,31,32]; limited evidence suggests that the addition of TSS might augment the therapeutic effects of AB-LT [33]. We observed modest improvements in the PBS, 10 MWT, and GMFM, with an increase in stride length, a decrease in stride length variability, and a decrease in cadence following AB-LT, suggesting improvements in gait efficiency at self-selected walking speeds. However, changes in stride width, time in stance and swing phase, and velocity were minimal or did not occur. In contrast, after AB-LT + TSS, larger improvements were seen in the 10 MWT and GMFM measures, and improvements were observed in nearly all spatiotemporal gait parameters at faster walking speeds. The improvements observed in this case study support the use of high-frequency and high-intensity activity-based interventions, as well as neuromodulatory techniques such as TSS, to promote meaningful improvements in the functional movement of children diagnosed with CP.

### 4.1. Improvements in Functional Movements and Balance

To show clinically significant improvement in gross motor function over time, the minimal clinical importance difference (MCID) for children with CP ranges from 0.1% to 3.0% for overall GMFM score [34]. The GMFM MCID value for dimension “D” ranges from 0.8% and 5.2%, and dimension “E” ranges from 0.3% and 4.9% [34]. After AB-LT alone, the Total GMFM score improved from 86.32% to 88%, and there was further improvement after AB-LT + TSS when the Total GMFM score improved to 93.7% (Table 2, Figure 2). The most noticeable improvement was seen in the Walking, Running, and Jumping dimensions (Dimension E), improving from 59.7% before AB-LT to 79.1% post-AB-LT + TSS. These results represent a remarkable improvement in the child’s ability to perform tasks related to independent walking. Both the Total GMFM score and the score for Walking, Running, and Jumping (Dimension E) increased well beyond their respective MCID values. This suggests that the AB-LT + TSS intervention had a greater impact on functional movement as measured by the GMFM.

A similar study was recently published in which 16 children with CP participated in a similar activities-based invention, including treadmill and overground training while applying TSS [33]. While this study only provided two sessions per week for 8 weeks, the authors also reported improvements in GMFM scores in all participants. However, this study did not incorporate a control group or sham conditions; thus, whether these functional improvements came from the activities-based intervention alone or the intervention in combination with TSS cannot be assessed. Our study therefore represents the first study to compare AB-LT with and without TSS in a child diagnosed with CP.

There was a small improvement in PBS scores over time. PBS scores improved from 40 (pre-AB-LT) to 42 (post-AB-LT) and to 44 (post-AB-LT + TSS) (Table 1). This may indicate that balance improvements were minimal as measured by the PBS. It is possible that the PBS is not sensitive enough to capture smaller gains in postural control. Another consideration is that the use of a harness during AB-LT and AB-LT + TSS intervention perhaps provided postural support, decreasing the postural challenge and interfering with potential postural control gains.

### 4.2. Improvements in Gait

Following AB-LT, notable improvements were observed in stride length, accompanied by a reduction in stride length variability (Table 1, Figure 3). This was likely due to the facilitation of increased step length and increased repetition provided during AB-LT, which has previously been shown to decrease performance variability [35]. The improvements in stride length and the reduction in variability persisted following AB-LT + TSS.

There was a clinically significant increase in velocity from before AB-LT to after AB-LT + TSS, as measured by the 10 MWT (Table 1). After AB-LT alone, there was a minimal increase in velocity from 1.05 m/s to 1.1 m/s, but a noticeable increase in velocity was seen following AB-LT + TSS when the velocity increased to 1.43 m/s. These results represent an important improvement in the child’s ability to walk at higher speeds after the AB-LT + TSS intervention.

Several spatiotemporal gait parameters improved following AB-LT and demonstrated further improvement following AB-LT + TSS (Table 1, Figure 3). The most important improvements in overground gait assessment at self-selected speed were seen in velocity and stride length. Other improvements were observed at fast walking speeds, with a considerable increase in velocity and stride length from pre-AB-LT to post-AB-LT + TSS. The improvements seen in gait parameters suggest a positive effect of AB-LT + TSS on the child’s overall ability to manage increased tone during gait, even when walking at a higher speed. Similarly, the step width increase observed may be an indication of the participant’s improved gait pattern, with decreased scissoring also likely due to better management of the dynamic increases tone observed during ambulation.

In addition to improvements seen in outcome measures, the participant and parent described positive functional changes occurring at home and in the community. The participant and his mother reported increased self-confidence and increased independence at home and in their community. For example, instead of sitting to prep meals, he started standing upright for a longer duration and could safely lift and carry large items across the kitchen. Additionally, he demonstrated improved strength and stability by carrying grocery bags from the car to the kitchen without stumbling. Furthermore, he achieved a significant milestone by navigating independently through a small-town rodeo without relying on any assistive devices.

### 4.3. GMFCS Classification

Before starting the AB-LT-only intervention, we classified the participant as GMFCS level III based on his and his mother’s reports and clinical observations [36]. There was no classification level change noted post-AB-LT only; however, we notably reclassified him at a level II post-AB-LT+TSS, based on participant and parent-reported level of function at home and in his community and direct observations. The participant reported walking without an AD more often and for longer distances in his community while continuing to carefully plan what type of mobility methods were safest, fastest, and the most energy-efficient [37]. We also considered environmental factors such as weather, types of terrain, distances traveled, and personal factors such as social participation in community events and social activities [37].

### 4.4. Mechanisms of Transcutaneous Spinal Stimulation (TSS)

Efforts have been made to activate the nervous system in children diagnosed with CP using central and peripheral electrical stimulation, leading to improved GMFM-88 scores in some participants [33]. Neuromodulation via TSS has been proposed to enhance the physiological states of spinal and possibly supraspinal networks, thereby improving coordinated movements [9]. Hasting et al. [33] proposed that spinal neuromodulation can suppress aberrant supraspinal connections, allowing children with CP to voluntarily initiate movements and gradually develop more typical proprioceptive patterns from enhanced spinal network signals [33,38]. This interaction between spinal motor output and proprioception may help diminish pathological supraspinal inputs, leading to improvements in posture and locomotion [19].

Additionally, TSS has been shown to alleviate various manifestations of spinal spasticity even after a single application [39,40]. The mechanism involves depolarizing proximal lower-extremity afferents, resulting in synchronized inputs to lumbar and upper sacral spinal circuits. This temporal relationship between postsynaptic activity and excitatory synaptic inputs—likely mediated by glutamate—further enhances intrinsic inhibitory mechanisms within the spinal cord. Thus, repetitive and synchronous neurotransmitter release can reduce excitatory postsynaptic potentials in target motoneurons, thereby increasing post-activation depression in spastic individuals [40].

In summary, the mechanisms underlying TSS suggest a promising approach for enhancing functional outcomes in children with CP. By modulating spinal and supraspinal networks, TSS can not only facilitate voluntary movement initiation but can also foster the development of typical proprioceptive patterns. The ability of TSS to alleviate spasticity while promoting coordinated movement further underscores its potential as a non-invasive intervention.

## 5. Limitations

Limitations include difficulty maintaining electrode connection with skin. Specifically, the presence of a harness over the area where the electrodes were applied, combined with the participant’s movement, resulted in multiple instances of electrode movement. Furthermore, the stimulator used did not allow for the use of TSS during overground ambulation due to its size. A more portable stimulator option is needed to allow for more versatile use of TSS during gait rehabilitation. This study is reporting the results of a single participant, which limits generalization to the CP population. Furthermore, the current study does not allow the analysis of whether the improvements noted were simply a cumulative result of an increased total number of AB-LT sessions. Though these results are promising, more children with CP need to be assessed using this protocol to determine its efficacy as a treatment option in this population.

## 6. Conclusions

This study demonstrates the clinical feasibility of integrating TSS into an AB-LT protocol for children with CP. The combination of TSS with AB-LT resulted in greater improvements in functional mobility and gait compared to AB-LT alone in an ambulatory child with CP. This finding suggests that activity-based interventions, when combined with neuromodulatory techniques such as TSS, may significantly enhance functional outcomes in ambulatory children with spastic CP. These findings highlight the potential of using targeted repetitive physical training (i.e., AB-LT) in conjunction with spinal stimulation to promote neuroplasticity and motor function improvement/recovery. This approach warrants further investigation through larger clinical trials to validate its efficacy in pediatric CP populations.

## Figures and Tables

**Table 1:** Outcome measure and spatio-temporal gait parameters results. PBS = pediatric. Balance scale. 10 MWT = 10 m walk test. GMFM = gross motor function scale—88. AB-LT = activities-based locomotor training. AB-LT + TSS = Activities-based locomotor training with transcutaneous spinal stimulation.

	Outcome Measures								
		PBS		10 MWT (m/s)		GMFM (%)			
		*x*/56	(%)	Self Selected	Fast	C	D	E	Total Score
	pre AB-LT	40.0	71.0	0.7	1.1	97.6	74.3	59.7	86.3
	post AB-LT	42.0	75.0	0.8	1.1	100.0	84.6	55.5	88.0
	post AB-LT + TSS	44.0	79.0	0.8	1.4	100.0	89.7	79.1	93.7
**Spatio-Temporal Gait Parameters**									
		**Stride Width**	**Stance (%)**		**Swing (%)**		**Stride Length**	**Velocity**	**Cadence**
		**(cm)**	**L**	**R**	**L**	**R**	**cm**	**(cm/s)**	**(Steps/min)**
self selected	pre AB-LT	6.2 ± 22.1	68.2 ± 9.8	68.0 ± 6.5	31.9 ± 9.8	32.0 ± 6.5	76.4 ± 18.4	70.2	114.0
	post AB-LT	6.9 ± 21.2	68.1 ± 3.7	67.8 ± 4.6	31.9 ± 3.7	32.2 ± 4.6	84.7 ± 9.6	72.4	103.0
	post AB-LT + TSS	21.5 ± 4.2	63.6 ± 3.8	71.0 ± 2.2	36.5 ± 3.8	29.0 ± 2.2	89.3 ± 7.9	76.7	103.9
fast	pre AB-LT	24.1 ± 4.2	61.5 ± 2.4	67.3 ± 1.4	38.5 ± 2.4	32.7 ± 1.4	102.0 ± 8.2	106.7	126.2
	post AB-LT	21.4 ± 2.9	63.0 ± 2.3	67.2 ± 2.1	37.0 ± 2.3	32.8 ± 2.1	102.7 ± 5.0	101.4	117.2
	post AB-LT + TSS	27.0 ± 3.3	55.3 ± 6.2	58.6 ± 4.71	44.7 ± 6.2	41.4 ± 4.7	113.7 ± 8.6	150.1	160.9

**Table 2:** Summary of activities-based locomotor training parameters. TM = treadmill. BWS = body-weight support. SR = step retraining. SA = step adaptability. STA = stand adaptability. Avg. = average. AB-LT = activities-based locomotor training. AB-LT + TSS = activities-based locomotor training with transcutaneous spinal stimulation.

BWSTT Session Data								
	Total TM Time	Avg. BWS	Total SR Time	SR Speed	Total SA Time	SA Speed	Total STA Time	Avg. Total Step Time
	(min)	%	(min)	(mph)	(min)	(mph)	(min)	(min)
AB-LT	52.97	35.53	15.22	1.2–1.8	11.64	1.2–1.5	25.22	26.86
AB-LT + TSS	56.70	28.25	20.13	1.6–2	12.75	1.4–1.8	22.23	32.88

## Data Availability

The original contributions presented in the study are included in the article; further inquiries can be directed to the corresponding author.
